# Oxidative and carbonaceous patterning of Si surface in an organic media by scanning probe lithography

**DOI:** 10.1186/1556-276X-8-75

**Published:** 2013-02-13

**Authors:** Matteo Lorenzoni, Andrea Giugni, Bruno Torre

**Affiliations:** 1 Nanophysics, Istituto Italiano di Tecnologia, Via Morego 30, 16163, Genova, Italy; 2 Nanostructures, Istituto Italiano di Tecnologia, Via Morego 30, 16163, Genova, Italy

**Keywords:** Scanning probe lithography, Nanocrystalline graphite, Dry etching mask

## Abstract

A simple top-down fabrication technique that involves scanning probe lithography on Si is presented. The writing procedure consists of a chemically selective patterning in mesitylene. Operating in an organic media is possible to perform local oxidation or solvent decomposition during the same pass by tuning the applied bias. The layer deposited with a positively biased tip with sub-100-nm lateral resolution consists of nanocrystalline graphite, as verified by Raman spectroscopy. The oxide pattern obtained in opposite polarization is later used as a mask for dry etching, showing a remarkable selectivity in SF_6_ plasma, to produce Si nanofeatured molds.

## Background

State-of-the-art technology in patterning semiconductor substrates mainly relies on mask-based techniques such as optical lithography or mask-less techniques like electron beam lithography, which, for their inherent multi-step and large area, parallel processing capabilities are particularly suited for industrial applications such as large numbers of device production in microelectronics and microfabrication in general. Aside some more flexible, fast, and easily modifiable processes, several scanning probe-related lithographies (SPLs) also emerged [1-3] as a research-oriented fast prototyping tool [4]. Nanofabrication by SPL is affordable and very versatile. The advantages of using an atomic force microscope reside in the nanometric accuracy in feature positioning and in the possibility of directly applying multistep processes on pre-patterned substrates with no need for alignment tools and/or photoresist coating.

This makes SPL an ideal tool for flexible and fast prototyping of custom nanodevices. Early studies were mainly focused on oxidation and reduction processes of Si and SiO_2_ to assess the capability to fabricate semiconductor-insulator nanojunctions, achieving a remarkable ultimate sub-10-nm resolution [5].

Besides the local oxidation of silicon, the dissociation of organic molecules under an intense electric field (approximately 10^9^ V m^−1^) localized below a biased AFM tip has been recently used to create nanometer-sized heterojunctions employing common organics [6,7] organometallics [8], or fluorinated solvents [9], obtaining remarkable results in terms of resolution (reaching 2-nm feature size), scalability (employing stamp technology) [5,6], and writing speed [7]. However, the technique is relatively new, and little effort has been made in extensively exploiting its wide fabrication capabilities.

**Figure 1:**
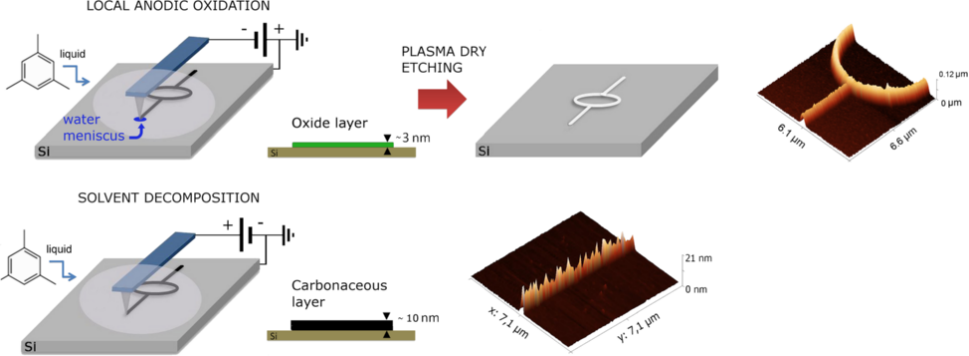
**Schematic of the fabrication steps.** AFM operates in contact mode in a liquid media (1,3,5-trimethylbenzene), depending on the bias applied; the deposited thin film is composed of silicon dioxide (local anodic oxidation) or graphitic carbon (solvent decomposition). In the case of oxidation, the pattern can be used as a mask for Si dry etching producing high aspect ratio features.

Suez and Rolandi showed how to shift from field-induced oxidation to solvent decomposition through silicon surface modification [10]; in this work, we present a simple fabrication technique that involves AFM top-down lithography that allows either oxidation or carbon deposition within the same pass. The writing procedure consists of alternating local anodic oxidation and solvent decomposition by controlling the tip's polarization (Figure 1). In short, oxidation occurs when a negative tip bias is applied while, applying a positive tip bias, the low volatility organic media is decomposed by high-field tip discharge occurring in a confined nanometric volume below the tip. The experiments were conducted in room environment with no need of temperature control. Features obtained in both polarities have a final lateral resolution below 60 nm and a voltage controllable single pass height. If oxide feature height ranges within what was previously reported [11] (1 to 4 nm) and shows a linear dependence with the bias applied, the carbonaceous features can reach heights above 40 nm and present slower growth rates. The choice of mesitylene as precursor is given by two reasons: on one side, this molecule has shown its capability to decompose under electric field, leaving pure *sp*^2^ carbon bodies [12]; it is therefore expected to leave pure *sp*^2^-clustered graphitic residuals if dissociated under a conductive probe. On the other side, due to its low volatility and relatively high vapor tension at room temperature (boiling point = 164.7°C), it can be dissociated in a liquid drop in ambient condition for hours, with no need of a closed liquid cell, trapping enough humidity to perform writing; it is therefore simpler to be used in multi-step processes. The solvent is drop-casted directly on the wafer (1 × 1 cm), and as the AFM tip approaches the surface, a liquid neck is formed between the surface and the holder.

**Figure 2:**
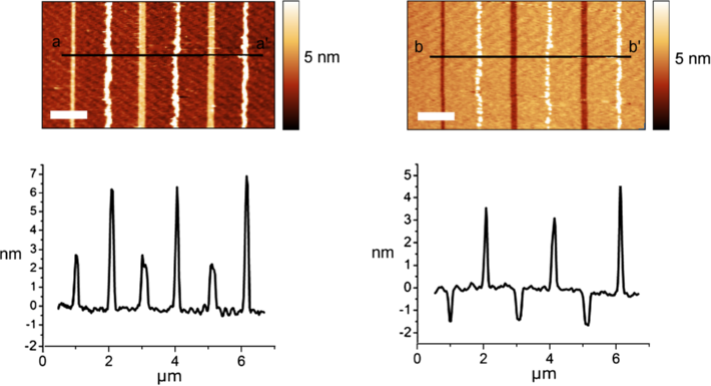
**Etching test on lines written alternatively by oxidation or carbon deposition.** On the left, AFM topography and height profiles of single lines written with opposite bias (±10-V tip bias, 0.5-μm s^−1^ writing speed). On the right, the same pattern after 1-min etching in aqueous HF 5 wt.%. The carbonaceous residual shows etch resistance while oxide is readily removed. Scale bare is 1 μm in both.

The following etch tests (Figure 2) led to the conclusion that with the solvent employed (mesitylene), oxidation takes place regardless of the surface wettability (Table 1); this is probably due to a higher content of water in mesitylene with respect to hexadecane. If we let anhydrous hexadecane (or similar hydrocarbons) in contact with humid air, the content of water dissolved in it will be approximately the same range of the water in air; more specifically, in the case of hexadecane, the molar concentration

$${\left(n/V\right)}_{H_{2}O/\text{hexadecane}}\approx 2.32\times 10^{-6}\mathrm{mol}/l$$,

and the molarity ratio of water in hexadecane to water in air at 25°C is 1.8 [13]. If we consider the chemically closest compound to mesitylene with available data on mutual solubility with water, we find that *p*-xylene at 25°C presents a water solubility of 440 ppm (alkenes range between 80 and 100 ppm) [14]. Under the same approximation made in [11], we calculated a molarity ratio of 16, meaning that the number of available water molecules in the mesitylene-like solvents is 16 times higher. Moreover, we concluded that the carbonaceous layer deposited consists of nanocrystalline graphite, as verified by Raman spectroscopy. The oxide patterns have been later used as etch resistant mask for http://inductively coupled plasma reactive ion etching (ICP-RIE) Si dry etching. Resulting Si 3D structures have single sub-100-nm-wide features up to 100-nm tall, thanks to a remarkably high selectivity to the SF_6_ plasma etchant used in the process, the same etching procedure did not produce satisfactory results on carbonaceous patterns.

**Table 1:** Water contact angles, height/bias dependence, and correlation coefficient for oxidation on different Si surfaces

**Surface termination**	**Contact angle of water droplet (°)**	**Slope (nm V**^**−1**^**)**	**Correlation coefficient (adjusted R**^**2**^**)**
Si(OH) native oxide layer	29 ± 0.9	0.40 ± 0.04	0.92
Si(H)^a^	81 ± 1.2	0.37 ± 0.01	0.99
Si(CH_3_)^b^	89 ± 0.8	0.48 ± 0.04	0.95

Data in Figure 4 have been used for linear fitting. At a constant writing speed (1 μm s^−1^), an increase of 1 V in bias produce a height increase of approximately 0.4 nm; ^a^30 s in aqueous HF 5 wt.%; ^b^hexamethyldisilizane vapors for 1 h in moderate vacuum.

## Methods

Polished p-type Si(100) wafers (resistivity 1 to 10 Ω cm) were sonicated for 10 min in acetone, ethanol, DI H_2_O immediately before processing, thus preserving a native SiO_2_ layer. The exposure of Si surface to a solution of aqueous HF (5 wt.% for 30 s) results in the removal of native oxide and surface H termination (water contact angle ≈ 80°). Silanization of Si(100) wafer has been achieved by exposing the surface, after degreasing, to hexamethyldisilizane (HDMS, ≥99%; Sigma-Aldrich Corporation, St. Louis, MO, USA) vapors for 1 h in moderate vacuum. The obtained wafers showed a water contact angle of approximately 90°. Depositions were performed with an Asylum MFP-3D (Asylum Research, Santa Barbara, CA, USA) operating in contact mode in liquid with integrated software to control lithographic parameters (Microangelo). The liquid environment (1,3,5-trimethylbenzene, ≥99.0%; Sigma-Aldrich) was exposed to typical ambient humidity (35% to 40%).

The probe employed during the fabrication tests was SiN Au-coated Olympus OMLC-RC 800 (*k* = 0.042 Nm^−1^, typical tip radius 430 nm), and the maximum bias applicable is ±20 V. It was possible to achieve a writing speed of 10 μm s^−1^, but the process is better controlled with a speed ranging from 0.2 to 5 μm s^−1^. Tip's wear does not compromise writing up to 10-mm continuous writing.

Raman spectra have been collected with a micro-Raman spectrometer Horiba T64000 (Edison, NJ, USA). Spectra have been recorded at room temperature, using an incoming laser light line linearly polarized at 514.5 nm from an Argon/Krypton ion laser (Ar/Kr Stabilite 2018-RM, Spectra-Physics, Mountain View, CA, USA), and a power density of about 2 mW μm^−2^ is used (×100 objective, Olympus SLM plan). The spectrometer resolution was determined by curve fitting the silicon 520 cm^−1^ band using a linear combination of Gaussian and Lorentian curves achieving full width at half maximum (FWHM) less than 2 cm^−1^. This silicon band was used for the precise calibration of energy scale.

Kelvin probe force microscopy measures have been performed with Asylum MFP-3D in air at room temperature (RH ≈ 35%) with Pt-coated probe Olympus OMCL-AC240TM. The work function of one reference tip (*Φ*_tip_ = 4.93 ± 0.05 eV) was calibrated by Kelvin probe force microscopy (KPFM) on freshly cleaved highly oriented pyrolytic graphite (HOPG).

Si dry etching was conducted with a Sentech ICP-RIE SI 500 plasma etcher (Sentech Instruments GmbH, Berlin, Germany). Working parameters for SF_6_ were as follows: gas flow 30 sccm, 1 Pa, RF/ICP power 600, and RF plate power 18 W. For pseudo Bosch (SiF_6_ + C_4_F_8_), gas SiF_6_ flow 30 sccm, C_4_F_8_ flow 32 sccm, 1 Pa, RF/ICP power 600, and RF plate power 18 W. Each sample has been finally cleaned by oxygen plasma. Fabricated masters have been imaged in tapping mode with standard Si cantilevers (Nanosensors PPP-NCH, Nanoworld AG, Neuchâtel, Switzerland; nominal resonant frequency *ca.* 330 kHz, force constant ≈ 42 Nm^−1^, polygon-based pyramidal tip with half cone angles of 20° to 30° with a tip apex radius below 10 nm). To minimize tip's convolution artifacts, some samples have been imaged using high aspect ratio tips (Nanosensors AR5-NCHR; nominal resonant frequency *ca.* 330 kHz, force constant ≈ 42 Nm^−1^) with half cone angle smaller than 2.8°. Energy diffraction spectroscopy (EDS) elemental analysis was performed by a X-Max large area analytical EDS silicon drift detector (Oxford Instruments, Oxford, UK) with (Mn Kα typically 125 eV) mounted on a JEOL 7500 FA SEM (Akishima, Tokyo, Japan).

## Results and discussion

**Figure 3:**
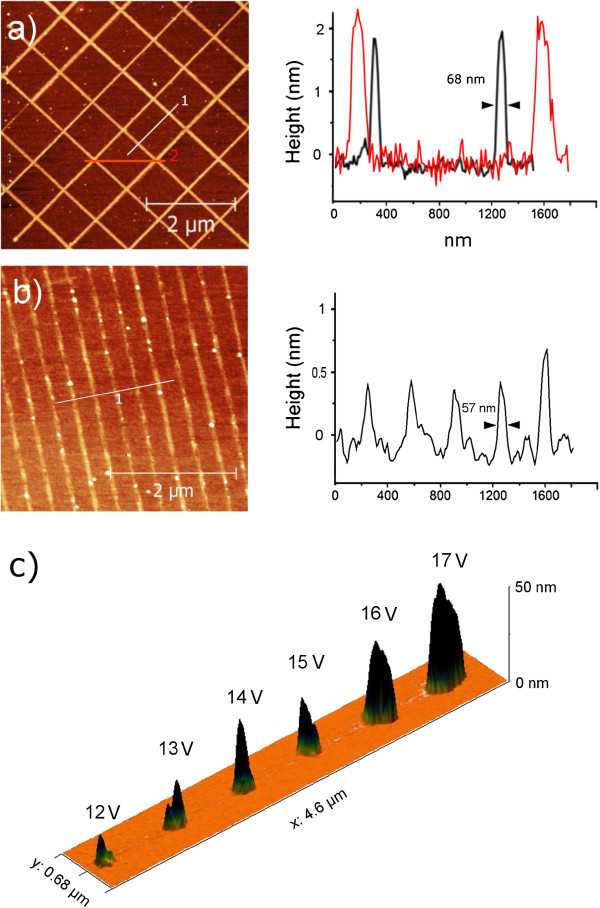
**Example of continuous patterns by oxidation or carbon deposition.** (**a**) AFM topography and height profiles of a grid with 750-nm spacing (−10-V tip bias, 5-μm s^−1^ writing speed) showing features with FWHM = 68 nm on Si(H). The points where two lines cross (red profile) show a slight increase in height (0.2 to 0.3 nm). (**b**) Parallel carbonaceous lines with 350-nm spacing (19-V tip bias and 1-μm s^−1^ writing speed). Average line height ≈ 0.5 nm, single feature FWHM = 57 nm. (**c**) Single carbonaceous spots deposited with a pulse of 0.5 s at increasing voltage; spot's height (>50 nm) is considerably higher if compared to oxidized spots (data not shown).

**Figure 4:**
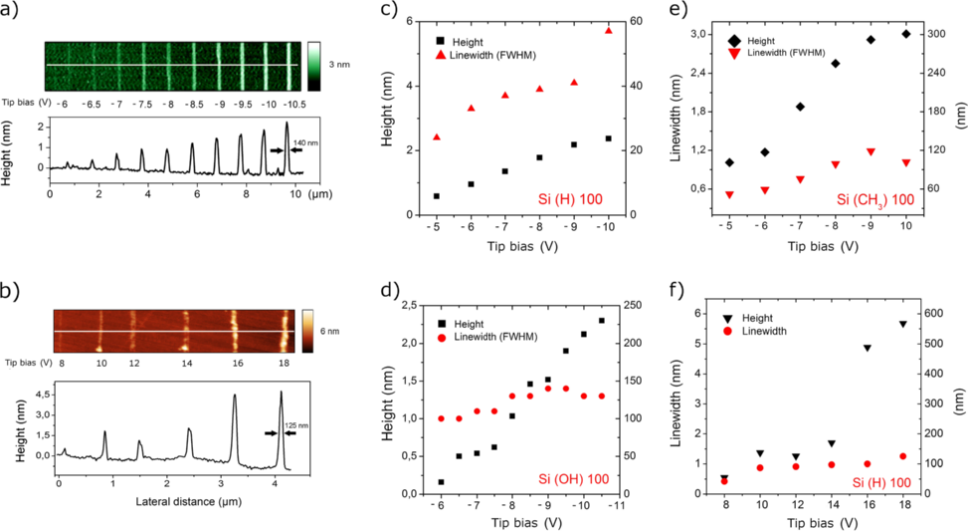
**Thickness and line width at various biases.** Height/bias dependence for oxide lines (**a**) and carbonaceous lines (**b**). AFM topographies and profiles refer to features written at 1 μm s^−1^. (**c** to **f**) Height/bias relation plotted for different Si surfaces, Si:OH or pristine (with native oxide layer), H-terminated, and methyl-terminated; for positive tip bias (carbonaceous), we show the Si(H) surface. Black marks refer to height, and red marks refer to the line width expressed as FWHM. The smallest lateral resolution (<40 nm) is achieved for oxide features on Si(H); similar line width is observed for Si(CH_3_), while as the surface becomes more hydrophilic, line width raises above 100 nm (**d**). As expected, oxide height (**c** to **e**) increases linearly with bias for all surfaces in the 5- to 11-V interval with a similar height/bias dependence. With a negative sample bias, the deposition still depends on voltage (**f**).

Writings have been realized in contact mode using SiN Au-coated probes with a tip radius <30 nm. The set-point force was maintained below 10 nN. As illustrated in Figure 1, applying a negative tip bias, Si oxidation takes place, thanks to the residual water molecules present in the solvent, the process is well controlled, confined by the meniscus size, and self limited due to the diffusion limit of oxidizing species through the grown oxide [11,15]. With a positive tip bias, the organic precursor is continuously dissociated under the AFM tip; the process, driven by the high electric field, involves a few tens of nanometers' area at the interface between the substrate and the tip apex. At a writing speed below 0.5 μm s^−1^ (Figure 2), a single line height of carbonaceous features approximately doubles the oxide height, increasing the writing speed to 5 μm s^−1^ (Figure 3); carbonaceous features' height drops to 0.5 nm. This is probably due to the different growth rates of the two processes, with and oxidation that is several orders of magnitude faster than the solvent decomposition. The different mechanism is also proved by the series of dots deposited with a pulse of 0.5 s at increasing voltage (Figure 3c), spot's height is considerably higher if compared to oxidation. As shown in Figure 4, at a constant writing speed (1 μm s^−1^), the feature height is tunable by controlling the bias applied for both processes (Figure 4a,b).

To clarify the solvent decomposition mechanism under a positively biased tip, further investigation is needed although the mechanism proposed by Vasko et al. [16], in our case involving electron tunneling from the substrate to the tip and formation of reaction intermediates, could provide a valid explanation.

Writing is successfully performed in both polarization on p-doped Si(100) wafers having three different surface terminations: H:Si(100), CH_3_:Si(100), and Si(100) with native oxide layer of 1.7 to 2 nm, as measured by ellipsometer (data not shown). The formation and the geometry of the water meniscus is ruled by a number of factors including capillary forces, electric field gradients, ambient humidity, as well as the wetting behavior of the substrate [17]. Oxide growth is confined by the water meniscus and thus sensitive to surface preparation that affects the capillary condensation at the water/silicon interface. As the surface becomes more hydrophilic, line width raises above 100 nm (Figure 4c,d,e) but is not inhibited. As water contact angle increases, the meniscus is likely to condense with different geometries resulting in narrower features (approximately 40 nm). Line height and width written by solvent decomposition (Figure 4f) still depend on the bias applied, but the non-linear behavior indicates a different undergoing mechanism with respect to local oxidation.

**Figure 5:**
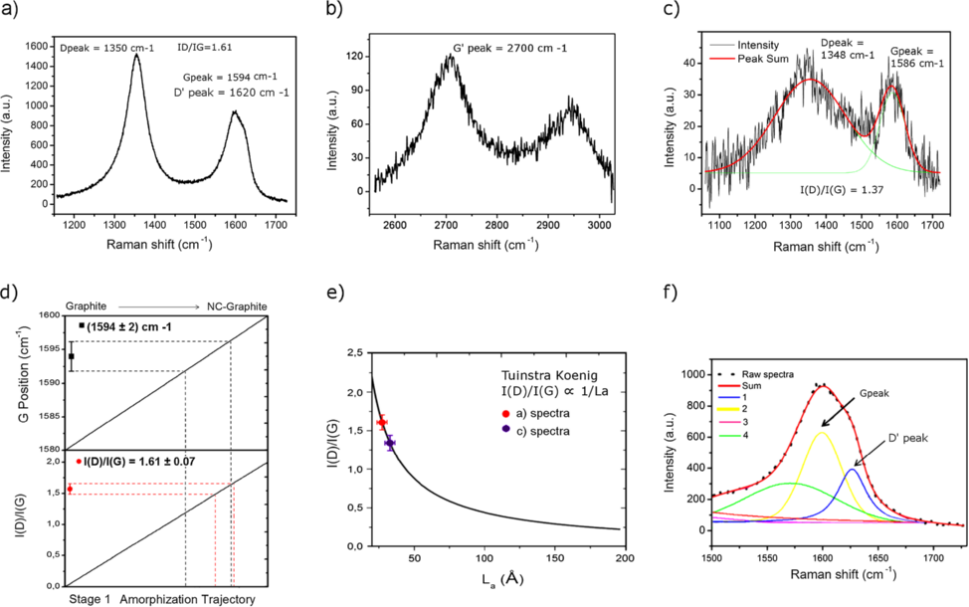
**Raman spectra of patterned regions.** (**a**, **b**) Two different spectral zones of the same sample patterned with a thicker carbonaceous layer (approximately 50 nm) while (**c**) spectra, bearing a lower signal, has been collected on a 2-nm-thick layer. All spectra have been fitted with a linear combination of Gaussian and Lorentzian curves (c, f) to extrapolate the peak centers. (**d**) The G positions and the *I*(G)/*I*(D) values fall within the theoretical first stage of the amorphization trajectory. (**e**) Interdefect distance *L*_a_ is deducted from Tuinstra-Koenig relation, valid for thin surface layers of a graphite sample. For details, see the main text. (**f**) A proposed fit that indicates the components of the band around 1,600 cm^−1^.

The carbonaceous composition of the deposit has been confirmed by EDS elemental analysis (see Additional file 1), while structural characterization has been performed by means of Raman spectroscopy and KPFM. Raman spectroscopy has been employed in order to assess the type of bonding present in the carbon deposited and its degree of amorphization. Detailed maps by micro-Raman spectrometer of two patterned areas were acquired with a Raman probe spot size of 41 μm (see Additional file 1). The Si background signal has been subtracted by the raw data. The average of nine highly representative spectra is shown in Figure 5a,b,c.

At a first glance (Figure 5), the sample shows the three intense Raman features also present in graphite and the two overlapping bands around 1,600 cm^−1^ present in nanographite [18] at approximately 1,595 cm^−1^ (G band), approximately 1,349 cm^−1^ (D band) and approximately 2,698 cm^−1^ (G′ band). According to the three-stage model of classification of disorder introduced by Ferrari and Robertson [19], the Raman spectrum is considered to depend on the degree of amorphization, the disorder, clustering of *sp*^2^ phase, presence of *sp*^2^ rings or chains, and ratio between *sp*^2^ and *sp*^3^ bonds.

The two parameters considered to identify the degree of amorphization are the G peak position and the *I*(D)/*I*(G) ratio, where *I* indicates the total intensity (i.e., area under the band). Assuming that the residual composition is homogeneous, we obtained a G position (*G*^POS^) = 1,594 ± 2 cm^−1^ and *I(D*)/*I*(G) = 1.61 ± 0.07 leading to the conclusion that the residual corresponds mainly to a graphite-like state with nanocrystalline structure, lying in between the so-called ‘stage 1’ in the amorphization trajectory (graphite → nanocrystalline graphite) presenting a negligible *sp*^3^ content and the ‘stage 2’ in which more defects appear together with a low *sp*^3^ content. In stage 1, the Tuinstra-Koenig [10] relationship links the interdefect distance *L*_a_ (and thus grain size) to the *I*(D)/*I*(G) ratio:

(1) $$\frac{I\left(D\right)}{I\left(G\right)}=\frac{C\left(\lambda \right)}{L_{a}}.$$

*C* constant depends on the wavelength; at 514.5 nm, its value is equal to 44 Å. Therefore from Equation 1, it is possible to estimate a grain size *L*_a_ = 36 ± 2 Å (Figure 5e). Our results are also consistent with a high content of *sp*^2^ hybridized carbon, as already reported by Suez et al. [10] for features deposited from a liquid aliphatic precursor (hexadecane). A more detailed evaluation of the band around 1,600 cm^−1^ (Figure 5f), by a multipeak fit, reveals that the three components could represent the sample spectra. The two components (G and D′) are present in the nanocrystalline graphite, and a third component around 1,570 (lowered G peak) is due to mainly *sp*^2^ amorphous carbon.

**Figure 6:**
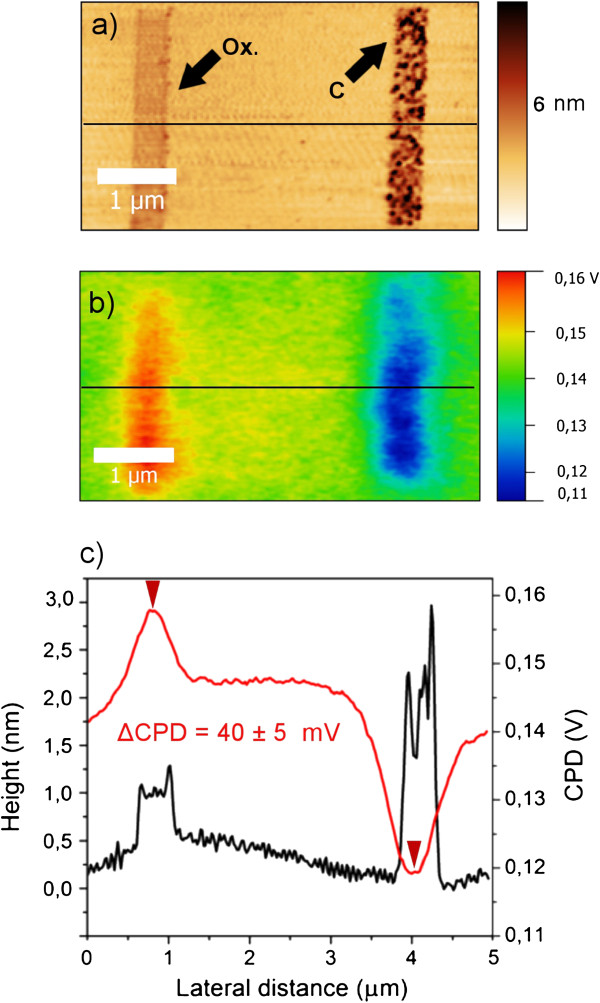
**AFM topography, KPFM scan, and comparison of height and CPD value profiles.** AFM topography (**a**) and KPFM scan (**b**) of a pattern made in both polarizations: oxide (left) and graphitic (right) body contours are clearly resolved by CPD difference. Comparison of height profile and CPD value profile (five-point average along the black line) (**c**).

Kelvin probe force microscopy measures local contact potential difference (CPD) between a conductive AFM tip and a sample. This difference is sensitive to local compositional and structural variations. The work function (*Φ*) of p-doped silicon(100) is ≈ 4.91 eV, and the work function of HOPG in air is ≈ 4.65 eV [20], the latter is used as reference. Based on those considerations, we expect a local drop in *Φ* where a graphitic layer is present and an opposite behavior in the presence of a dielectric layer (SiO_2_). We performed CPD scan over both patterns, and the findings are presented in Figure 6, showing the expected local CPD behavior. During the scan, we applied an AC voltage dithering the tip at a frequency of 79 kHz. In order to avoid artifacts, trace and retrace data were always collected and compared. Topography and potential were collected simultaneously performing a so-called NAP scan at a constant height of 40 nm. The work function of one reference tip (*Φ*_tip_ = 4.93 ± 0.05 eV) was calibrated by KPFM on freshly cleaved HOPG. The relative CP difference within the single scan gives the most reliable information; due to unavoidable environmental pollution (adsorbate layers) and *Φ*_tip_ variability among different probes even within the same session, the reproducibility of CPD absolute values is critical. Considering that *Φ*_sample_ = *Φ*_tip_ − eV_CPD_, we obtained:

$$\begin{array}{l} \;\Phi_{{\mathrm{SiO}}_{2}\;\mathrm{pattern}}=4.93\pm 0.05\;\mathrm{eV}-0.16\;\mathrm{eV}=4.77\pm 0.05\;\mathrm{eV}\\ \;\Phi_{\text{C}\;\mathrm{pattern}}=4.93\pm 0.05\;\mathrm{eV}-0.12\;\mathrm{eV}=4.81\pm 0.05\;\mathrm{eV} \end{array}$$

The difference in work function measured allows to clearly resolve patterned graphitic bodies and partially confirms the prevalent graphitic composition of the features although it was not possible to get a quantitative explanation of the local work functions measured.

**Figure 7:**
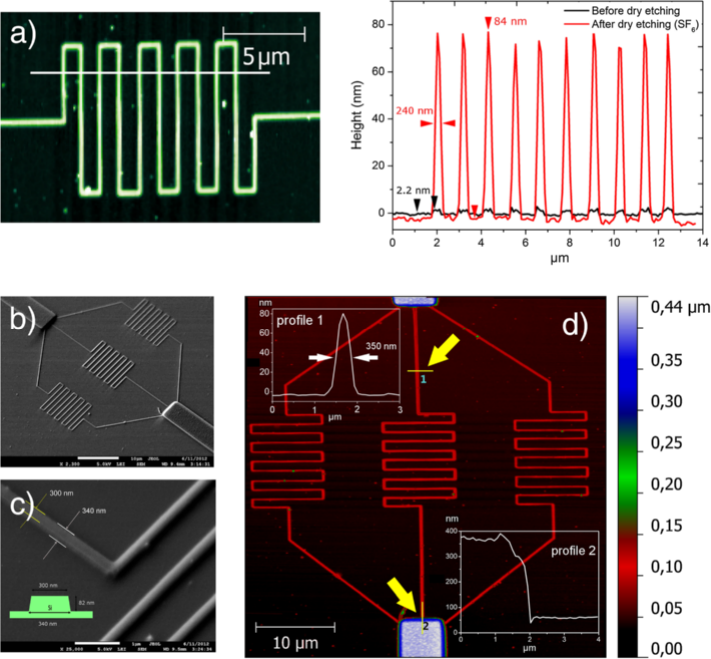
**Example of finalized prototype.** (**a**) AFM topography of multiple line pattern written at a 2-μm s^−1^ speed and a bias of 12 V used as mask for an 8-s etching in SF_6_ plasma; on the right, the height profiles before RIE (black) and after RIE (red). (**b**, **c**) SEM images showing the finalized result of fabrication; in the details, the effective size and section of features are available. (**d**) AFM topography of a finalized Si prototype; Al microfeatures are connected to nanofeatures deposited by SPL. Profile 1 shows the obtained section, and section 2 shows the junction profile (no gap is observed).

The use of fluorocarbon resist patterns fabricated by SPL as mask for silicon dry plasma etching has been already reported [6]. Due to the better control achieved through oxidation in this work, we tested standard silicon dry etching only on fabricated oxide patterns. The plasma gases employed were a SF_6_ and SF_6_/C_4_F_8_ (pseudo Bosch). Exposure times ranged from 5 to 30 s. The different etch rate between Si substrate and oxide features result in a gain in features' height. A maximum enhancement (final and initial average height ratio ≈ 40:1) occurs after 8 s of exposure to SF_6_ (Figure 7a), while pseudo Bosch plasma quickly consumes the mask, and the ratio between final and initial average height remains constant around 5:1 for different etching times. We calculated an etch rate of 22 nm min^−1^ leading to a selectivity ≈ 42 over p-doped Si(100), relative to a measured attack rate of SF_6_ over Si of 940 nm min^−1^. Those values are compatible with what was reported for SF_6_ dry etching of wet and dry oxides. The etch rate is slightly influenced by several factors: single lines resist less than dense areas patterned by multiple lines, higher voltages during lithography produce features more resistant to etching, and any shape defect produced during deposition will affect the etching process. Imaging of grooves and protrusions can be affected by artifacts. A tip with a relatively large cone angle overestimate the real width of steep vertical features and fails to penetrate into deep and narrow grooves. That error is negligible for thin films as-deposited but is maximized for features with rectangular section between 50- and 100-nm tall; in order to minimize such effect for the topographies, we used a high aspect ratio tip. To prove the potentiality of the process, we prepared a Si mold intended for nanofluidic applications (Figure 7); to verify that we can create junctions between micro- and nanostructures, we fabricated aluminum micropatterns (approximately 300-nm thick) by vapor deposition with a conventional masking made by laser writing. This mold consisted in three nanocircuits connecting a 40 micron interruption left in between the line; those microfeatures once transferred on soft replica (i.e., PDMS) represent the access channels to lower scale nanochannels (see Additional file 1 for examples of fabricated PDMS replica). The gaps have been successfully connected with the fabricated structure showing a continuous pattern as shown in the profile 2 of Figure 7d.

## Conclusions

We illustrated a simple and inexpensive nanofabrication method that can produce oxide or pure graphitic nanofeatures by means of SPL, employing almost any commercial AFM, avoiding subtractive fabrication methods including electron beam lithography and focused ion beam. Secondly, choosing a proper organic precursor, we show that the technique is accessible to most AFM users with no need of dedicated setups in ambient environment. The reaction leading to carbon deposition is likely to happen in both polarities, but when the tip is biased negatively, the competing oxidation masks solvent decomposition. The method, combined with dry etching allows the fast prototyping of Si masters ideal for replica molding/nanoimprinting. As a possible prototype, we realized several Si masters with satisfactory aspect ratio and we showed how to hybridize microlithography with SPL, connecting Al micropatterns with nanopatterns.

## Competing interests

The authors declare that they have no competing interests.

## Authors’ contributions

ML carried out the experiments, prepared the samples, and wrote the manuscript. BT supervised the work and helped during the experimental design and discussion of the results. AG performed the Raman characterization. All authors read and approved the final manuscript.

## Supplementary Material

Additional file 1 **Oxidative and carbonaceous patterning of Si surface in an organic media by scanning probe lithography.** The file contains experimental details (Figures S1 and S2) and supplementary examples of fabrication capabilities (Figures S3 to S5). Click here for file
