# Supplementary material for: Oxidative and carbonaceous patterning of Si surface in an organic media by scanning probe lithography
Source: Nanoscale Res Lett. 2013 Feb 13;8(1):75. doi: 10.1186/1556-276X-8-75 (PMC3576257; doi:10.1186/1556-276X-8-75)
Supplement: Additional file 1 — Oxidative and carbonaceous patterning of Si surface in an organic media by scanning probe lithography. The file contains experimental details (Figures S1 and S2) and supplementary examples of fabrication capabilities (Figures S3 to S5). [file 1556-276X-8-75-S1.docx]

**Oxidative and carbonaceous patterning of Si surface in an organic media by scanning probe lithography**

Matteo Lorenzoni*, Andrea Giugni and Bruno Torre

***Nanophysics, Istituto Italiano di Tecnologia, Via Morego 30, 16163 Genova (Italy); +39 010 71781856 [matteo.lorenzoni@iit.it](mailto:matteo.lorenzoni@iit.it)

Supplementary information


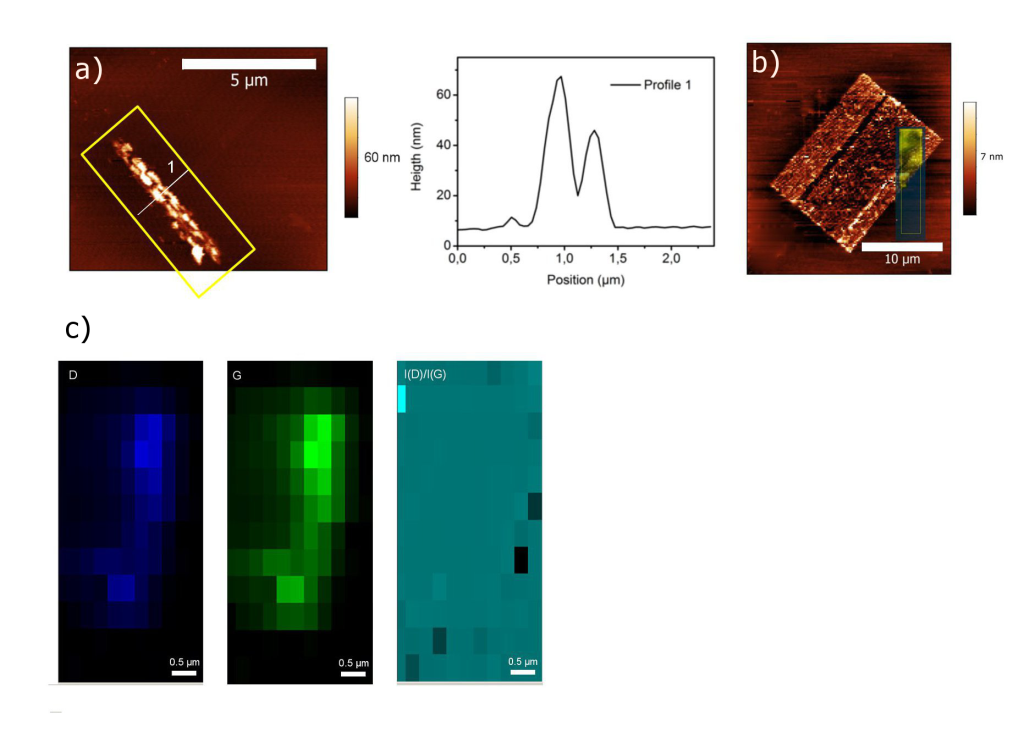


Figure S 1 – Patterns used for Raman characterization. In (a) AFM topography and height profile of the thicker carbonaceous layer. The pattern was obtained by overlapping several lines spaced 50 nm written with 18V tip bias, 1 µm s ^-1^ speed. The yellow rectangle indicates the area used to perform Raman color mapping (c). I(D)/I(G) ratio is constant on the all pattern. In (b) AFM topography of a pattern with a thinner graphitic layer (2 nm) and an highlight of the area were the Raman signal was acquired.


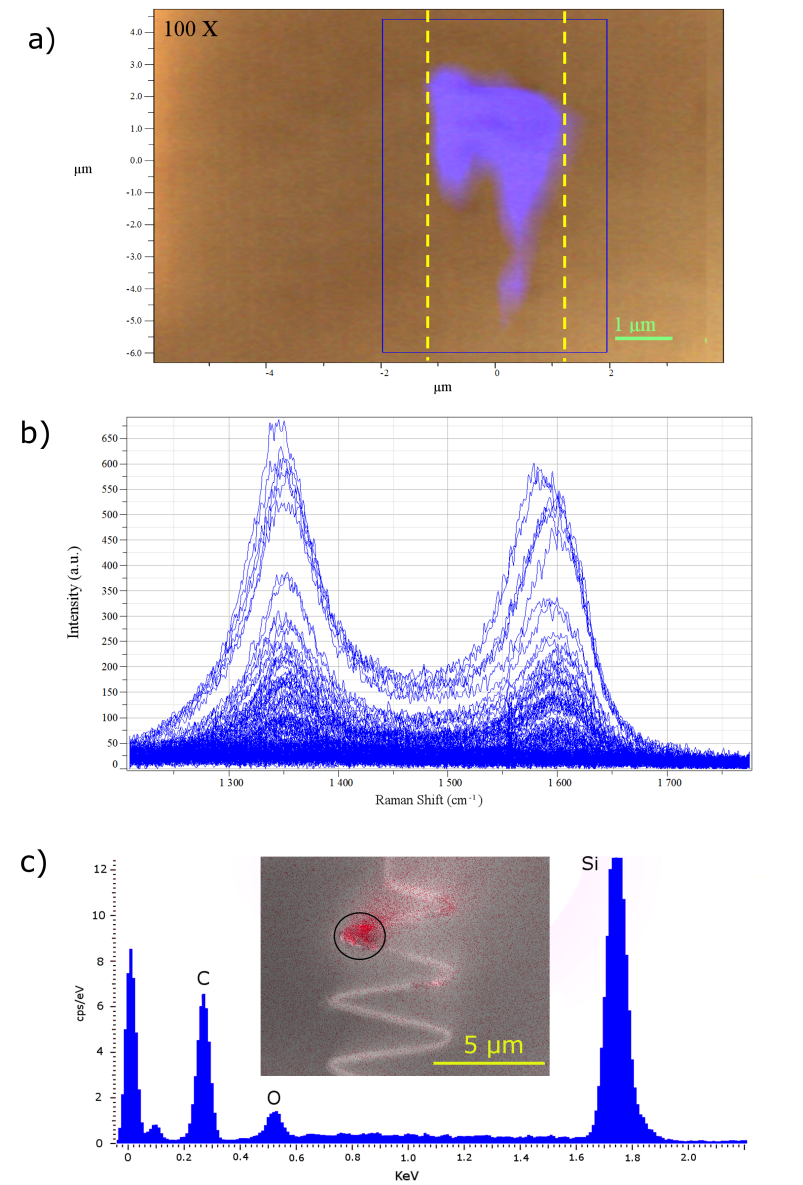


Figure S 2 - EDS chemical map superposed to SEM image of a sample written at 14V and 0.5 µm s ^-1^ speed, C is mapped with red, the spectra refer to the area circled in black. C presence is confirmed.

**
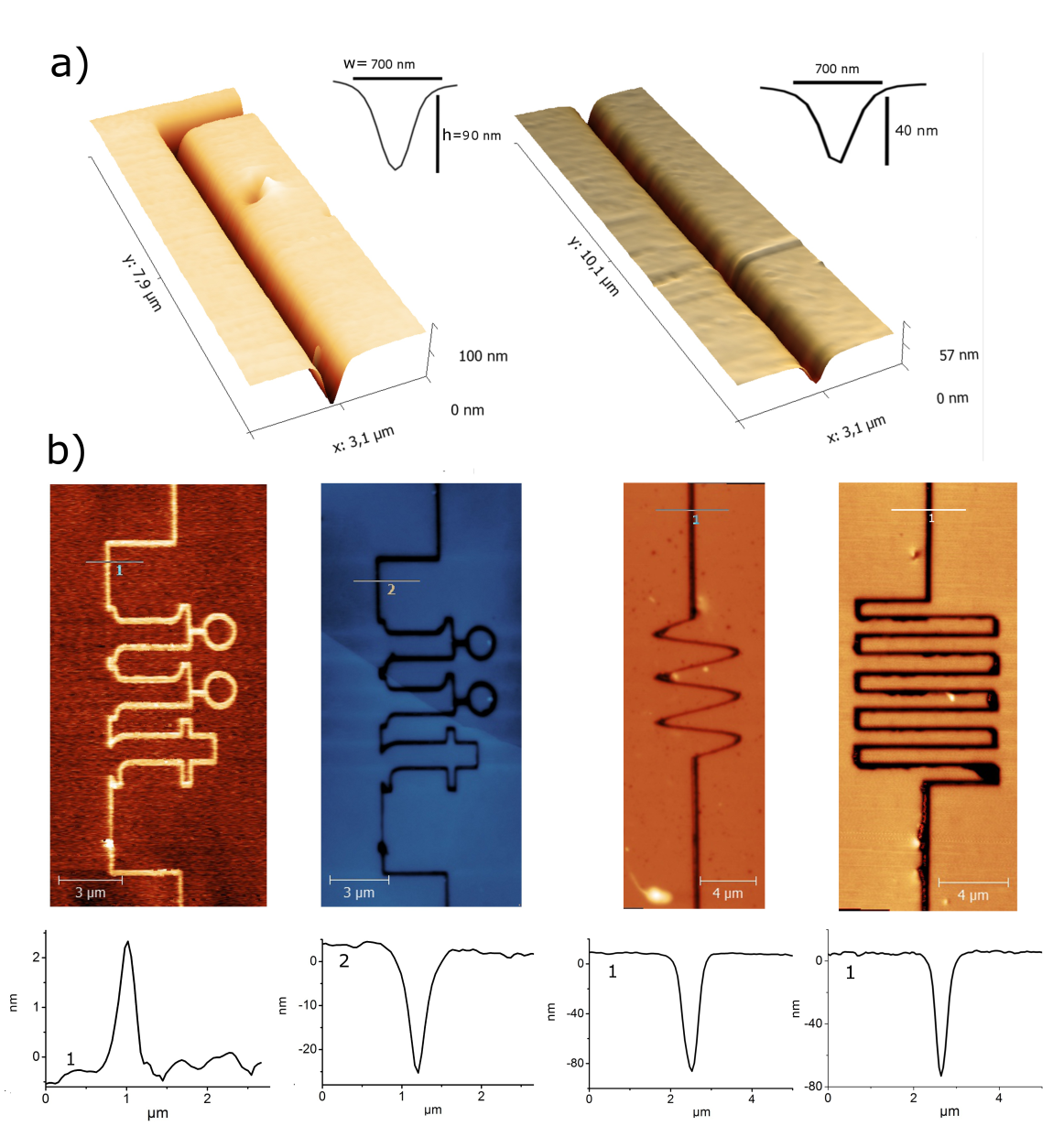
**

Fig. S3 - Examples of obtained PDMS replica: (a) AFM topography 3D images and section of channels with different aspect ratio obtainable. The real height of channels is underestimated due to difficulties in imaging deep trenches during scan with standard aspect ratio tips; In (b) comparison between the starting feature (i) and final PDMS replica obtained (ii); (j) and (k) are topography of two more nanochannels tentative.


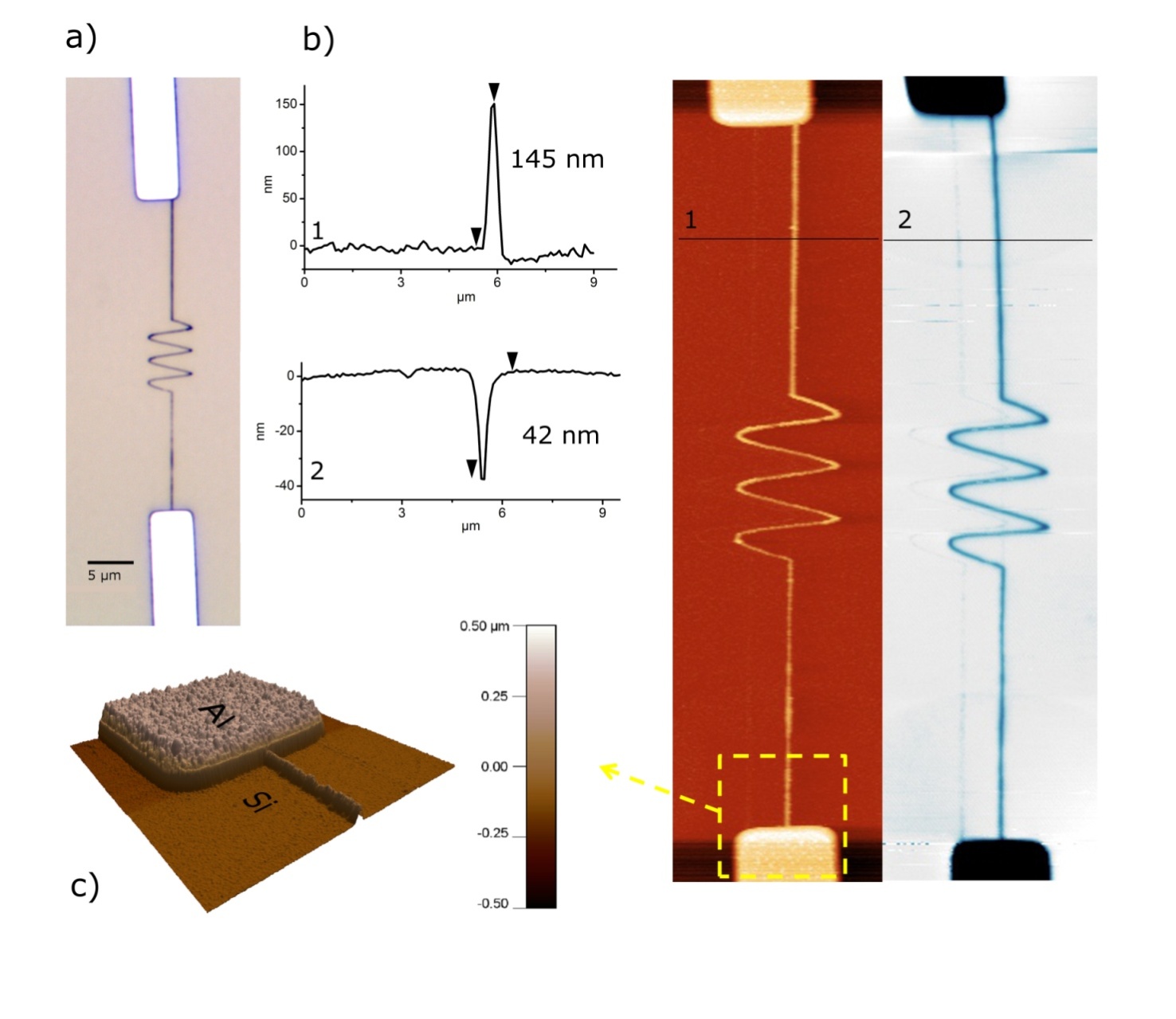


Fig. S4 - In (a) an image of the structure taken at the optical microscope (100X magnification). In (b) AFM topography of a supplementary example of master and the PDMS replica obtained. In (c) 3D detail of the junction area between Al and Si.


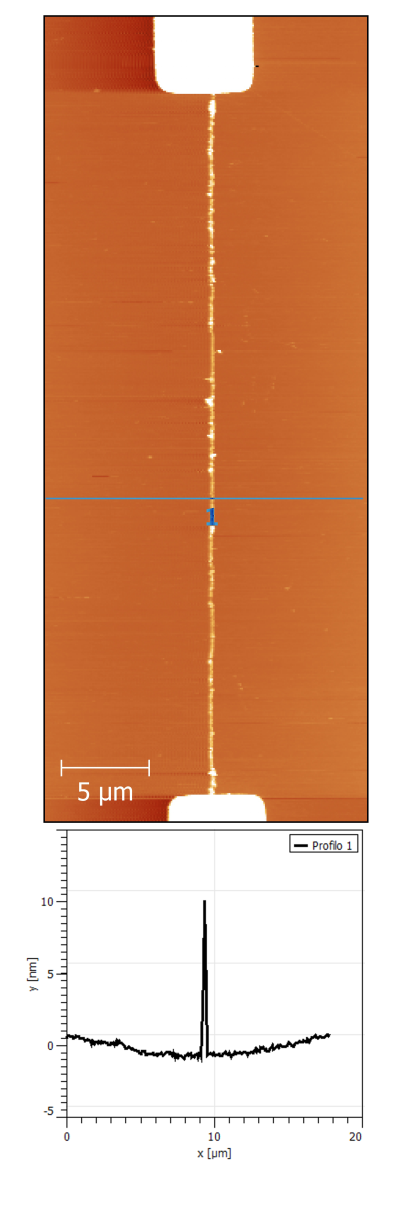


Fig. S5 – Single graphitic wire connecting two Al pads deposited on Si(100) p doped.
